# *Aronia melanocarpa* Products and By-Products for Health and Nutrition: A Review

**DOI:** 10.3390/antiox10071052

**Published:** 2021-06-29

**Authors:** Tomislav Jurendić, Mario Ščetar

**Affiliations:** 1 Bioquanta Ltd. for Research and Development, Trg Zlate Bartl 11/A, 48000 Koprivnica, Croatia; 2 Faculty of Food Technology and Biotechnology, University of Zagreb, Pierottijeva 6, 10000 Zagreb, Croatia; mscetar@pbf.hr

**Keywords:** *Aronia melanocarpa*, nutrition, polyphenolic compounds, antioxidant activity, health benefits

## Abstract

Due to factors such as cultivar, fertilization, maturation or climate conditions, as well as the date of their harvest, chokeberries (*Aronia melanocarpa*) differ in their content of minerals, vitamins, carbohydrates, amino acids, organic acids, fats, aroma compounds and especially polyphenols, substances exerting a beneficial impact on health. The total content of the most important ingredients, polyphenolic compounds, influence many proven chokeberry activities like antioxidative, anti-inflammatory, hypotensive, antiviral, anticancer, antiplatelet, antidiabetic and antiatherosclerotic, respectively. Polyphenolic compounds such as anthocyanins, flavonoids, procyanidins and phenolic acids in different rates and amounts are responsible for all mentioned activities. In the human body, they undergo different biotransformative processes strengthening their bioactivity inside and outside cells. The popularity of chokeberry has been significant lately because of its effects on human health and not just because of its nutritional value. The main interest in this review has been refocused on the chokeberry benefits to human health, nutritional contribution of its components, particularly polyphenolic compounds, and its physiological effects.

## 1. Introduction

The medicinal use of herbs in the prevention and treatment of diverse diseases is an old practice that has been maintained over time and is currently being given special attention by researchers and consumers [1]. Chokeberry (*Aronia melanocarpa*) belongs to the Rosaceae family and originates from the eastern parts of North America [2]. Chokeberries were highly valued and utilized by Native Americans to make tea to cure colds and the bark was used as an astringent [3,4].

Two species of the *Aronia* genus can be distinguished: *Aronia melanocarpa* (Michx.) Elliot known as black chokeberry and *Aronia arbutifolia* (L.) Pers. (red chokeberry), whereas the third entity is a hybrid of the two mentioned species, called *Aronia prunifolia*, purple chokeberry [4]. Since its strong resistance to cold, the crop can be grown not only in milder climate conditions but also at temperatures below −35 °C [5]. The *Aronia* shrubs grow to a maximum height of 2–3 m and have umbels of 20–30 small white flowers, from May to June, that ripen into black berries 6–13 mm in diameter, weighing 0.5–2 g [6]. Because of the sour taste and astringent properties, the chokeberry pomes are rarely used in direct consumption as natural fresh fruits [2]. Chokeberries became popular mainly for the large-scale production of juices, jams, wines, liqueurs and schnapps [4].

Due to the presence and the high content of various bioactive components, such as vitamins, minerals and polyphenolic compounds, the chokeberry and leaves of *Aronia melanocarpa* exhibit a wide range of positive health effects [7,8]. Along with their high antioxidant capacity, the *Aronia melanocarpa’s* main polyphenolic components also possess anti-inflammatory, anticancer, antimicrobial, antiviral, antidiabetic, antiatherosclerotic, hypotensive, antiplatelet and anti-inflammatory properties [2,3,4,7,8]. Polyphenols have been suggested to play a preventive role in the development of cancer and heart disease [9]. The black, dark violet and red color fruits are widely recognized as a valuable source of anthocyanins, one of the most widespread families of natural pigments in the plant kingdom and are, therefore used as a safe and natural food colorant with pro-health properties [6,10].

Because of the very complex intermolecular interactions, in addition to many human health promotion properties, further studies on chokeberry compounds and their benefits are required. The aim of this work is to review the nutritional profile and chemical composition of *Aronia melanocarpa* berries, products, by-products and leaves to emphasize their beneficial health properties.

## 2. Chokeberries Nutritional Profile

Chokeberries contain numerous compounds such as carbohydrates, organic acids, amino acids, minerals, vitamins, aroma compounds and polyphenols [4]. The chemical composition of chokeberry fruit depends on many factors including, climate conditions, soil composition, berry maturity, harvest methods and storage conditions, and significantly differs from other fruits with higher amounts of polyphenols [11]. Polyphenols are carriers of a characteristic flavor, smell, color, nutritive value and antioxidative activity [12].

The overall composition of *Aronia melanocarpa* products and by-products, respectively chokeberry, juice and pomace are shown in Table 1. The data specified in Table 1 and Table 2 indicate that the contribution of *Aronia melanocarpa* to the recommended daily intake of essential compounds is slight. However, its importance, with respect to nutrition, lies in the manifold physiological effects [7].

### 2.1. Dietary Fiber

Dietary fibers were determined as very important compounds of food.

An adequate intake for total fiber was set at 25–40 g per day [18].

The *Aronia melanocarpa* berries contained dietary fiber amounting to 5.6 g/100 g FW [4] where fiber content in pomace varies from 63% to 78% DM [15]. Data presented by [19] showed that the dietary fiber of chokeberry pomace is characterized by a substantial content of celluloses (35%), hemicelluloses (34%), lignin (24%) and pectin (8%). *Aronia melanocarpa* by-products rich in dietary fiber are considered a source of valuable ingredients for food supplements and functional foods [18].

Dietary fibers in the body show the possibility of sorbing harmful substances and thus, bind heavy metals and mineral components thereby, reducing their levels (e.g., cholesterol) [19].

### 2.2. Fat

The total fat content of berries was analyzed to be 0.14 g/100 g FW, where the largest amount was found in stones and skin fractions [4]. The content of fat in pomace amounted to between 3% and 14% DM, where the seed fractions of pomace were the richest in fat content [15]. According to [20] the seeds contained 19.3 g/kg FW glyceride oil. The oil obtained from *Aronia melanocarpa* was shown to be rich in phospholipids, sterols and tocopherols [20]. The fatty acids composition of dried pomace and seeds was characterized by a high content of polyunsaturated fatty acids (73.6% of total fatty acids) with linoleic acid as the main fatty acid [20,21].

### 2.3. Organic Acids

The presence and amounts of organic acids strongly affect food acceptance. Chokeberries have a generally low organic acid content which varies between 1.1% to 1.4% [22,23]. The main acids identified in fresh berries were L-malic (13.1 g/kg), citric (2.1 g/kg) and quinic (5.9 g/kg) acid. Shikimic acid, oxalic acid, and succinic acid were found as minor components [4,23]. The content of free acids in pomace is low because they transfer to the juice with other soluble substances. According to [15], among the organic acids in pomace galacturonic acid is dominant (5–16 g/kg).

### 2.4. Proteins and Amino Acids

The amount of protein in the fruit was low and amounted to 0.7 g/100 g FW [4]. Total protein content in dried pomace varied between 5% and 24%, whereas glutamic acid, aspartic acid and arginine were the most abundant amino acids [21]. It was also found that the seedless fraction of pomace possessed a remarkably lower content of protein than the seed fraction [15,21].

### 2.5. Sugar

Sugars are the main carbohydrates in *Aronia melanocarpa* while the total sugar content may vary between 68 and 158 g/kg of FW [24]. The studies by [14] pointed out that the content of glucose ranges between 11–40 g/kg, fructose 14–42 g/kg and sorbitol 44–76 g/kg of FW. Sucrose was detected in berries in amounts up to 0.34% [23]. Saccharide composition of fruit pomace may reflect the intensity of the juice manufacturing process which is often connected with enzymatic processes as well as an additional stage of pomace extraction after the first pressing [15]. The total sugar content in pomace was found to be 84 g/kg while the pomace was characterized by a lower content of sugar compounds (2.7–3.5% DM) with sorbitol (sugar alcohol) as the dominant component. It was also mentioned that seed fractions of pomace possessed a substantially higher content of saccharose and glucose than the seedless fractions. A daily 100 g dose of berries will cover about 3% of the required daily energy (Table 2).

### 2.6. Vitamins, Minerals and Trace Compounds

The amounts of vitamins and minerals in chokeberries and their contribution to the recommended daily intake is shown in Table 2. Freshly squeezed juice contains vitamins such as B1 (25–90 µg/100 mL), B2 (25–110 µg/100 mL), B6 (30–85 µg/100 mL), ascorbic acid (5–100 mg/100 mL), pantothenic acid (50–380 µg/100 mL) and niacin (100–550 g/100 mL). The mineral content (ash values) of fresh chokeberry was found to be 4–6 g/kg [4], while the content in juice was 5 g/kg [16]. The total content of ash in the pomace was at the level of 1.4–3.9% in DM, where the seeds had the highest content of ash [15].

Authors [25] reported that the dominating macro-elements of the fresh chokeberry, juice, pomace and leaves are potassium and calcium. Furthermore, the importance of macro-elements responsible for the control and regulation of metabolism was particularly emphasized. Otherwise, micro-elements play important biological roles as an integral part of enzymes or protein structures and are involved in electron transport, oxygen storage, redox processes, metal transport and biochemical processes [26]. The major micro-elements found in dried pomace were Fe, Zn and Cu [15], while by-products were additionally found to contain Mn and Sn [26]. The macro- and micro-element content in chokeberries, juice, pomace and leaves are represented in Table 3.

### 2.7. Aroma Components

In *Aronia melanocarpa,* more than 48 volatile compounds were identified, where the most important regarding quantity is amygdalin, and others like benzaldehyde cyanohydrin, hydrocyanic acid, benzaldehyde, benzyl alcohol, 2-phenylathanol, phenylacetaldehyde and many others [4]. The main compounds of juice aroma according to [26] belong to the following chemical groups: alcohols (48.9%), ketones (30.3%), hydrocarbons (0.2%), acids (5.8%), aldehydes (2.9%), terpenes (0.6%), esters (0.3%) and others (1.3%). The most abundant compounds in juice were 3-penthen-2-one (23.6%), 1-hexanol (18.2%) and 2-hexen-1-ol (11.1%) according to [26]. Amygdalin, a cyanogenic glycoside is responsible for the bitter-almond smell of the fresh fruit [4]. It dominates mostly in seed fractions; therefore, the elimination of seeds from pomace during the production of dietary fiber and polyphenolic preparations is necessary [27]. Cyanogenic compounds present in plants in the form of glycosides can be harmful because of releasing hydrogen cyanide when chewed and digested [27].

### 2.8. Polyphenols

Polyphenols are the most important antioxidants in the human diet [11]. Chokeberries are some of the richest sources of polyphenols among other berry fruits [28] and are high in content of procyanidins, anthocyanidins and phenolic acids, while flavonols are present in low amounts [9]. Researchers [29] report that the polyphenolic composition of the chokeberry significantly changes during fruit development and ripening, where the highest content of total polyphenols was observed for unripe fruit. Although processing influences the phenolic content of final products reaching consumers [30], it was found that *Aronia melanocarpa* products contain high amounts of polyphenols [31]. Authors [11] showed how weather conditions, such as temperature and insolation, influenced phenolic content in the juice. It was found that warm and dry climate conditions have a positive impact on the increasing value of total phenolics. The total polyphenol content in pomace falls into the range from 3100 to 6300 mg/100 g DM [14] and higher in seedless fractions of pomace when compared to the fractions with seeds [15]. Polyphenols were also found in leaves. According to [8], the total phenolic content of leaf extracts can vary between 1946 and 9148 mg/100 g and was higher in samples of leaves harvested at a more mature stage. Frequent consumption of dietary components such as polyphenols is desirable and is in line with the advice to eat five or more servings of fruits and vegetables a day. It is currently difficult to recommend exactly what doses of specific polyphenols should be consumed through foods in order to achieve the maximum benefits [32]. The average intake of phenolics is estimated at 1 g per day [33]. Table 4 shows the major phenolic phytochemicals present in berries, juice, pomace and leaves, with some considered significant chokeberry markers. The most significant marker compounds in chokeberries are anthocyanins and quercetin derivates; their chemical structures are represented in Table 5 and Table 6.

### 2.9. Procyanidins

Polymeric procyanidins were identified as the major class of polyphenolic compounds and represent 66% of the fruit’s polyphenols. Polymeric flavan-3-ols are composed predominantly of (−)-epicatechin as constitutive units of procyanidins connected mainly with C4-C6 and C4-C8 bonds [5,9]. Research shows that almost 40% of the antioxidant activity of chokeberries is attributable to procyanidins [34]. The size of procyanidin molecules can be described by their degree of polymerization (DP) [13]. The free compound epicatechin is also present in black chokeberries, only its concentration is significantly lower compared to polymeric procyanidins [35]. The highest content of total procyanidins was observed for unripe fruits, while the content declined during fruit development [29]. In the pomace, the content of polymeric procyanidins was found to be higher than in juice and fresh chokeberries [9]. Research by authors [15] reported higher contents of procyanidins in the seedless fractions of pomace. Procyanidins, among other phenolic compounds, impart the astringent effect while consuming the fruit [9,29].

### 2.10. Anthocyanins

Chokeberries are one of the richest plant sources of anthocyanins [4]. Anthocyanins in *Aronia* represent about 25% of total polyphenols. They are mainly a mixture of four different cyanidin glycosides: 3-*O*-galactoside (68.9%), 3-*O*-glucoside (1.3%), 3-*O*-arabinoside (27.5%) and 3-*O*-xyloside (2.3%). Furthermore, amounts of pelargonidin 3-*O*-galactoside and pelargonidine arabinoside were detected in trace [9]. The total content of anthocyanins can vary between 307 and 1480 mg/100 g FW, respectively [4]. In the research of [29], the fraction of anthocyanins was 41% of total polyphenols, which was much higher compared to the fraction in red raspberry (19%) and strawberry (23%). Moreover, the increase of anthocyanin concentration during ripening enhances the color and visual attractiveness of the fruit [29]. During processing of fruits, the total quantity of anthocyanins can be significantly lower [32]. The leaves of *Aronia* species were shown to contain low amounts of anthocyanin. The total content of anthocyanins detected in leaves was less than 2 mg/100 g DW [8].

### 2.11. Phenolic Acids

Phenolic acids represent 7.5% of chokeberry polyphenols and the main compound found in fruits was chlorogenic acid. It was found that the concentration of phenolic acids in juice (808.9 mg/100 g DW) was higher than in pomace (373.6 mg/100 g DW) which indicated its good solubility in water [9]. The predominant compounds in all leaf extracts from all three *Aronia* species were chlorogenic and neochlorogenic acids, while in the leaves of *Aronia arbutifolia* high amounts of rosmarinic acid were estimated [8].

### 2.12. Flavonols

Many studies showed that chokeberries have a low content of flavonols, around 1.3% of total polyphenols. Most of it was quercetin, while the most abundant are glycosides including 3-*O*-rutinoside, 3-*O*-galactoside and 3-*O*-glucoside [9]. Quercetin-3-*O*-vicianoside as well as quercetin-3-robinobioside were present in trace quantities [6]. The analysis of [8] showed that the total flavonol content in leaves (180–786 mg/100 g DW) was higher than in fruits (12–44 mg/100 g DW).

**Table 5:** Chemical structures of anthocyanins [3,36].

Phenolic Constituents	Substituents			Chemical Structure
Cyanidin-3-*O*-galactoside	R_1_ = galactose	R_2_ = OH	R_3_ = OH	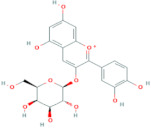
Cyanidin-3-*O*-glucoside	R_1_ = glucose	R_2_ = OH	R_3_ = OH	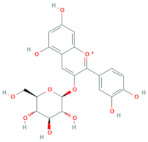
Cyanidin-3-*O*-xyloside	R_1_ = xylose	R_2_ = OH	R_3_ = OH	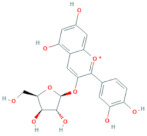
Cyanidin-3-*O*-arabinoside	R_1_ = arabinose	R_2_ = OH	R_3_ = OH	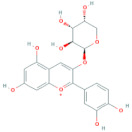
Pelargonidin-3-*O*-arabinoside	R_1_ = arabinose	R_2_ = H	R_3_ = OH	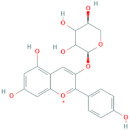
Pelargonidin-3-*O*-galactoside	R_1_ = galactose	R_2_ = H	R_3_ = OH	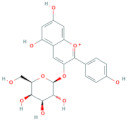

**Table 6:** Chemical structures of quercetin glycosides [3,24,36].

Phenolic Constituents	Substituents	Chemical Structures
Quercetin-3-*O*-vicianoside	R = vicianose	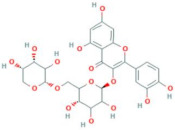
Quercetin-3-*O*-robinobioside	R = robinose	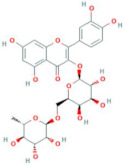
Quercetin-3-*O*-rutinoside	R = rutinose	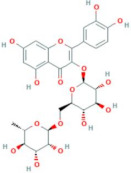
Quercetin-3-*O*-galactoside	R = galactose	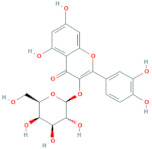
Quercetin-3-*O*-glucoside	R = glucose	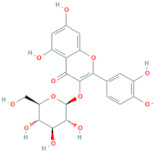
Quercetin-3-arabinoside	R = arabinose	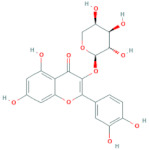
Quercetin-3-*O*-glucuronide	R = glucuronide	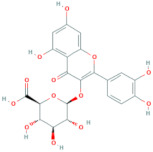
Quercetin-3-*O*-xyloside	R = Xylose	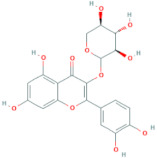
Quercetin-3-*O*-arabinoglucoside	R = arabinoglucoside	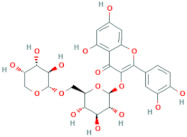
Quercetin-3-*O*-(6′-malonyl)-glucoside	R = malonyl-glucoside	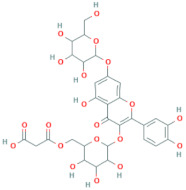

## 3. Beneficial Health Properties of Chokeberry Fruits

### 3.1. Antioxidant Effects

Regular consumption of fruits, vegetables and other food rich in antioxidants is often associated with improvement in overall health and lower incidences of chronic diseases [5]. With over 8000 known representatives in the plant kingdom, polyphenols are the most abundant and important dietary antioxidants [5,25]. The high content of polyphenols is responsible for the strong antioxidant properties of chokeberries and their products [11]. A consequence of the redox potential of phenols is their antioxidant action, where hydrogen donors and singlet oxygen quenchers allow them to act as reducing agents with the potential for metal chelation [9]. All these properties allow them to act against oxidative stress and to exhibit strong protective effects against cellular oxidative damage [9].

In order to evaluate the antioxidant activity of *Aronia melanocarpa*, the most commonly used assays include the inhibition of DPPH (2,2-diphenyl-1-picrylhydrazyl) and ABTS (2,2-azino-bis (3-ethylbenzothiazoline-6-sulfonate)) radicals [9]. Chokeberries show one of the strongest in vitro radical scavenging activities among other berries [28]. The measurement of ability to scavenge DPPH and ABTS radicals [9] revealed notable variation between antioxidant activities (all expressed as radical equivalents per µM Trolox/100 g DW) of fresh fruit (279.38 for DPPH and 439.49 for ABTS radicals), juice (127.45 for DPPH and 314.05 for ABTS radicals) and pomace (301.89 for DPPH and 779.58 for ABTS radicals). The in vivo mechanisms of antioxidant activity of phenols themselves, after absorption, extend far beyond radical removal including, suppression of reactive nitrogen (RNS) and oxygen species (ROS), recovery of antioxidant enzymes, inhibition of prooxidants and cellular signaling to regulate antioxidant levels and enzymes [25,31].

Cyanidin-3-*O*-arabinoside possesses the strongest radical-scavenging properties among the anthocyanins present in *Aronia melanocarpa*, and it was shown to be a strong inhibitor of pro-oxidative enzymes, like 15-lipooxygenase and xanthine oxidase [35]. Quercetin showed the highest oxygen radical absorbance capacity (ORAC) and total radical-trapping antioxidant parameter (TRAP) antioxidant activity in fresh chokeberries, but due to the low quantity of flavonols in fresh chokeberries, their contribution to the antioxidant activity was shown to be less than 10% [25]. It was demonstrated that 40% of the in vitro antioxidant activity of chokeberries is due to the potency of proanthocyanidins, followed by anthocyanins (24%), hydroxycinnamic acids (18%) and epicatechin (11%). Procyanidins are considered superior antioxidants compared to their corresponding monomers [13]. The green, unripe chokeberries have the highest antioxidant activity due to the high content of procyanidins and flavonoids, in spite of the absence of anthocyanins [29]. The results obtained by [8] showed that leaves of the *Aronia* species also possess a strong antioxidant capacity and are of potential therapeutic and dietary interest. [37] reported that the daily consumption of 150 mL of juice by rowers performing physical exercise during a 1-month training camp decreased the exercise-induced oxidative damage to the red blood cells.

### 3.2. Anti-Inflammatory Activity

The anti-inflammatory properties of *Aronia melanocarpa* fruit are related to the prevention of the development of chronic diseases, such as diabetes, cardiovascular diseases and chronic problems with the immune system [7]. Cyclooxygenases (COXs) and inducible nitric oxide synthase (iNOS) are the key pro-inflammatory enzymes responsible for the synthesis of lipid mediators and nitric oxide, associated with the progression of many inflammatory diseases [10]. The study by [38] showed an anti-inflammatory activity of *Aronia melanocarpa* extract on endotoxin-induced uveitis in rats. The in vitro experiment indicates that anti-ocular inflammatory action may involve inhibition of nitric oxide, prostaglandin, and tumor necrosis factor-α (TNF-α) production, resulting from suppressed expression of iNOS and COX-2-enzymes. The study by [39] demonstrated new evidence that the extract can inhibit the pro-inflammatory response of human aortic endothelial cells. [40] provide the first experimental support for the therapeutic application of the bioactive fraction against various inflammatory airway disorders. Besides the decreased expression of iNOS and COX-2, their study provided clear evidence for the anti-inflammatory activity through attenuation of ROS secretion and induced cell cycle arrest.

### 3.3. Antidiabetic Activity

Chokeberries are a good choice for the treatment of diabetes because they effectively improve glucose metabolism [7]. As well, antidiabetic activity was established for chokeberries as well as for leaf extracts, often in animal models with experimentally induced diabetes [3]. Chokeberry juice administered perorally for 6 weeks significantly reduced plasma glucose levels in rats with streptozotocin-induced diabetes [41]. Research by [16] suggested that juice suppressed the elevation of postprandial blood glucose levels through inhibition of dipeptidyl peptidase IV, α-glucosidase and angiotensin-converting enzymes, involved in the regulation of carbohydrate metabolism and the development of diabetes, respectively.

### 3.4. Antibacterial Activity

Since the flavonoids are known to be synthesized in plants in response to microbial infection, it should not be surprising that they have been found in vitro to be effective antimicrobial substances against a wide variety of microorganisms [7]. Polyphenols are very effective antimicrobial components in berry crops [7], and the berry constituents have an inhibitory effect on biofilm formation [42]. Chokeberry extracts exhibited bacteriostatic activity in vitro against *Staphylococcus aureus* and *Escherichia coli* [42]. The microbial activity test against 10 different pathogens showed that proanthocyanidins were the most potent antimicrobial agents [25]. According to [42] the leaf extracts showed an inhibitory effect on the growth of *Bacillus cereus*.

### 3.5. Antiviral Activity

Researchers [43] reported the antiviral activity of chokeberry juice. It was demonstrated that fruit juice inhibited the reproduction of the influenza virus in its initial stages so that the formation of complex compounds between the virion and polyphenol inhibited the adsorption of the influenza virus on the cell. Study [44] showed that *Aronia melanocarpa* possess in vitro and in vivo efficacy against different subtypes of influenza viruses. It is assumed that anti-influenza properties were attributed to two polyphenolic constituents, ellagic acid and myricetin. Recently, [45] indicated that a wide variety of plant species contain biologically active substances, especially polyphenols, which in synergistic combinations are effective in combating various diseases and are natural inhibitors of viral enzymes. Quercetin and ellagic acid, besides other phytochemicals, combined with the virus proteins showed potential antiviral activity against SARS-CoV-2 [45].

### 3.6. Antimutagenic and Anticancer Activity

Authors [33] reported a 60% growth inhibition of human HT-29 colon cancer cells after a 24-h exposure to anthocyanin extract without affecting the growth of normal cells in vitro. The treated cells showed a blockage at G1/G0 and G2/M phases of the cell cycle. The comparative study showed that *Aronia melanocarpa* extract inhibited growth to a greater extent than grape and bilberry anthocyanin-rich extracts when inhibition was compared to a similar concentration of anthocyanins. The leaf extract also showed anticancer activity through inhibition of SK-Hep1 human hepatoma cell growth and metastasis of cancer cells [46]. Ref. [47] indicated that the polyphenol-rich *Aronia melanocarpa* juice effectively and selectively induced programmed cell death of T cell-derived lymphoblastic leukemia cells. It was assumed that *Aronia melanocarpa* polyphenols regulate the expression of key regulators of G_2_/M cell cycle transition and apoptosis. The anticancer activity was associated predominantly with chlorogenic acids, some cyanidin glycosides, and derivates of quercetin.

### 3.7. Cardiovascular Disorder

Hypertension is the major contributor to cardiovascular and related disease development, associated with endothelial dysfunction and oxidative stress [5,48]. Regular juice drinking resulted in the reduction of total cholesterol, LDL (low-density lipoprotein) cholesterol and triglycerides levels and induced a significant decrease in the systolic and diastolic blood pressure in men with mild hypercholesterolemia [49]. Polyphenols can affect general cardiovascular health and hypertension, because of their ability to reduce vascular oxidative stress. The results of studies in rats with induced hypertension show reduced blood pressure values in the study group treated with the ethanolic extract of *Aronia melanocarpa* compared with the control group [48]. In the same study, they emphasized that the drop in blood pressure was provoked by an improvement in total antioxidant capacity and a decrease in lipid peroxidation. In vivo and in vitro experiments demonstrate that the phenolic constituents contribute to the protection and restoration of endothelial cells [50] as well as anti-platelet effects [51,52]. Since it has been shown that oxidative stress can contribute to the pathogenesis of cardiovascular diseases, the intake of dietary antioxidants should be recommended for their prevention [5].

### 3.8. Hepatoprotective Activity

Hepatoprotective activity of *Aronia melanocarpa* juice was investigated in experiments in rats with carbon tetrachloride CCl_4_-induced acute liver damage, where the addition of juice to the diet of rats prior to CCl_4_ treatment significantly reduced histopathological changes in the liver [53]. The protective effect is in great measure related to its antioxidative properties and the scavenging of free radicals formed during CCl_4_ intoxication. Results obtained by [54] indicated that *Aronia melanocarpa* anthocyanins have anti-fibrotic effects on CCl_4_-induced liver injury in mice, whereas anthocyanins showed the ability to inhibit the transforming growth factor-β signaling pathway and reducing the expression of inflammatory factors such as TNF-α (tumor necrosis factor α) and interleukin-1 (IL-1). According to [55], the use of anthocyanins resulted in a decrease of cadmium in cadmium-accumulated liver and kidneys of rats. [56] showed that the supplementation with berry extract significantly prevented Cd-mediated changes in the expression of collagen types I and III, as well as deregulation of the matrix metalloproteinases/tissue inhibitors system in the liver. The study of [57] confirmed that 6-week *Aronia melanocarpa* administration can alleviate liver damage in mice induced by 24-week alcohol feeding through inhibition of oxidative stress and reduction of inflammatory reactions.

### 3.9. Overweight and Obesity

Overweight and obesity are defined as abnormal or excessive fat accumulation that may impair health. In 2019, 38.2 million children under the age of 5 were overweight or obese [58]. Overweight and obesity are the major risk factors for noncommunicable diseases, especially diabetes and cardiovascular diseases [2]. *Aronia melanocarpa* extract-treated high fat diet (HFD)-induced obese mice showed significant decreases in body weight, serum triglyceride, and LDL cholesterol levels and improved insulin sensitivity as compared with HFD controls [59]. Study [60] reported an inhibitory activity of polyphenols, especially proanthocyanidins, against pancreatic lipase what refers to the suppression of dietary fat absorption and to a strategy against overweight and obesity.

### 3.10. Other Health Problems

Research [61] reported that *Aronia melanocarpa* extract intake regulates thermogenesis in healthy women with a cold constitution. It was suggested that *Aronia melanocarpa* intake improves the maintenance of body temperature through the regulation of noradrenalin and oxidative stress levels. The leaf extracts showed an acetylcholinesterase and butyrylcholinesterase inhibition rate of around 60–70%, which could be important in the development of natural materials as a treatment for neurodegenerative disorders such as Alzheimer’s disease, Parkinson’s disease and dementia [42]. Many in vitro and in vivo experiments indicate that extract and juice from chokeberries and their leaves may protect from toxic effects connected with pro-oxidative and pro-inflammatory properties of some drugs, tobacco smoke and its components, radiation and other xenobiotics [2].

## 4. Conclusions

The main nutrition- and health-relevant *Aronia melanocarpa* components are polyphenols, sugars, minerals and vitamins. Many in vitro and in vivo investigations confirm these substances are producing numerous nutritional and physiological activities like antioxidative, anti-inflammatory, hypotensive, antiviral, anticancer, antiplatelet, antidiabetic and antiatherosclerotic. *Aronia melanocarpa* products and by-products require a wide range of studies to further confirm their safety, efficacy and stated mechanism of action, especially in the nowadays very popular antiviral activities. A better understanding of the role of *Aronia melanocarpa* products and by-products in human nutrition and their contribution to human health could be of great importance, as well. The data existing in the literature demonstrate the potential of *Aronia melanocarpa* as a nutritionally rich and healthy dietary food with many functionalities and benefits.

## Figures and Tables

**Table 1:** Chemical composition of chokeberry, juice and pomace.

Chemical Composition	Berries g/kg	References	Juice g/L	References	Pomace g/kg	References
Dry matter %	15–31	[6,13]	11–17	[14]	45–50	[14]
pH	3.3–3.7	[4]				
Titratable acidity (g citric acid per 100 g)	0.5–1	[6,13]	0.9–1	[11]	0.5–0.6	[15]
Total sugar	68–158	[14]	110–143	[14]	84	[14]
Glucose	11–40		32–40		22	
Fructose	14–42		30–39		24	
Sorbitol	44–76		48–64		38	
Total polyphenols (Chromatographic method)	79	[9]	4.7–9.0	[14]	31–63	[14]
Fiber	56	[4]	3	[16]	630–780	[15]
Minerals	4–6		5		14–39	
Fat	1.4		<1		29–139	
Proteins	7		2		49–241	
Amygdalin mg/100 g	20	[15]	5.8	[15]	7–185	[15]

**Table 2:** Chokeberry Nutrients and Recommended Daily Intake.

Ingredient	Amount in 100 g [4]	Recommended Daily Intake [17]				Percent Daily Value
		1–4 Years Old	4–15 Years Old	After 15 Years Old	Average	%
Energy (kcal)						
Carbohydrates (Kcal)	60	1100–1300	1300–2900	2000–3400	2000	3
Proteins (g)	0.7	14	18–50	48–67	40	2
Fats (g)	0.14	-	-	-	-	
Minerals (mg)						
Sodium (Na)	2.6	400	500–1400	1500	950	0.3
Calcium (Ca)	32	600	750–1200	1200	900	4
Potassium (K)	218	1100	1300–3600	4000	2550	9
Magnesium (Mg)	16	80	120–310	350–400	240	7
Zinc (Zn)	0.2	3	4–12	11–16	10	2
Iron (Fe)	0.9	8	8–15	12–15	12	8
Vitamins (mg)						
Thiamin (B1)	0.02	0.6	0.7–1.2	1.1–1.4	1	2
Riboflavin (B2)	0.02	0.7	0.8–1.4	1.2–1.6	1	2
Pyridoxin (B6)	0.03	0.6	0.7–1.5	1.4–1.6	1	3
Niacin	0.3	8	9–15	13–17	12	3
Phantothenic acid	0.3	4	4–6	6	5	6
Ascorbic acid (C)	14	20	30–85	90–110	65	22
Tocopherols (E)	1.7	5–6	8–14	12–15	10	17
Folate/µg	20	120	140–300	300	210	10
Phyllochinon (K)/µg	24	15	20–50	60–80	48	50

**Table 3:** Macro- and micro-element contents (mg/100 g) in *Aronia melanocarpa* berries, juice, pomace and leaves.

Elements	Berries [25]	Juice [25]	Pomace [15]	Leaves [25]
K	271–498	85–320	181–308	262
Ca	60–117	14–123	219–408	373
P	24–96	17–104	239	151
Mg	16–58	21–59	37–250	83
Na	1–2	2–6	5–9	2
Zn	0.4–0.8	0.1–0.3	0.6–3.7	1.2
Fe	0.9–1.4	0.7–2.5	7.5–8.6	2.2
Se	0.02	0.07–0.1		0.05
Cu	0.08–0.2	0.1–0.5	0.5–1.2	0.4
Mo	0.002	0.005–0.006		0.008
Cr	0.05	0.06–0.07		0.05
Mn	0.5–1.8	0.3–1.2	3.2	0.6
Si	0.2–0.6	0.3–0.7		0.6
Ni	0.01–0.07	0.01–0.09		0.01
B	0.3–1.4	0.1–0.9		0.5
V	0.04–0.2	0.1		0.2
Pb	0.005–0.009	0.006–0.01		0.005
Cd	0.02–0.004	0.005–0.006		0.002
As	0.03–0.04	0.06–0.08		0.03

**Table 4:** Phenolic phytochemicals present in *Aronia* fruit, pomace, juice and leaves.

Phenolic Constituents	Fruit mg/100 g DW [8,9]	Fruit mg/100 g FW [6,13,29]	Pomace mg/100 g DW [9]	Juice mg/100 g DW [9]	Leaves mg/100 g DW [8]
**Flavan-3-ol**					
(−)-Epicatechin	15	32	11	13	
Procyanidins	5182	1646	8192	1579	
Degree polymerization (DP)	23	59	34	23	
**Anthocyanins**					
Cyanidin-3-*O*-galactoside	19–1282	417–636	1120	787	0.2–2
Cyanidin-3-*O*-glucoside	0.3–42	8–27	79	28	
Cyanidin-3-*O*-arabinoside	6.2–582	129–299	533	324	0.2
Cyanidin-3-*O*-xyloside	53	29–38	105	34	
**Phenolic acids**					
Chlorogenic acid	16–302	72–111	204	416	184–706
Neochlorogenic acid	92–291	59–100	169	393	143–483
3,4-Dihydroxyphenylacetic acid	4–26				5,8–66
Protocatechuic acid	0.4–31				2–9
Rosmarinic acid	9–18				23–155
**Flavonols**					
Quercetin	12–44	7.1			83–316
Quercetin-3-*O*-galactoside	37	7–13	47	50	
Quercetin-3-*O*-glucoside	22	4	27	31	
Quercetin-3-*O*-rutinoside	15	4	14	28	62–103
Quercetin-3-*O*-rhamnoside					97–367
Quercetin-3-*O*-vicianoside		3–5			
Quercetin-3-*O*-robinobioside		1–5			
Quercetin derivates unidentified	27		82	47	
Kaempferol		0,5			
**Flavanon**					
Eriodictyol-7-*O*-glucuronide		24			

DW: dry weight; FW: fresh weight.

## Abbreviations

ABTS	2,2-azino-bis(3-ethylbenzothiazoline-6-sulfonate)
CCl_4_	Carbon tetrachloride
COXs	Cyclooxygenases
DPPH	2,2-diphenyl-1-picrylhydrazyl
DM	Dry matter
DP	Degree of polymerization
DW	Dry weight
FW	Fresh weight
HFD	High fat diet
IL-1	Interleukin-1
LDL	Low-density lipoprotein
iNOS	Inducible nitric oxide synthetase
ORAC	Oxygen radical absorbance capacity
RNS	Reactive nitrogen species
ROS	Reactive oxygen species
TNF-α	Tumor necrosis factor α
TRAP	Total radical-trapping antioxidant parameters
